# Valorizing okara waste into nutritionally rich polysaccharide/protein-extracts for co-encapsulation of β-carotene and ferrous sulphate as a potential approach to tackle micronutrient malnutrition

**DOI:** 10.1016/j.jff.2021.104749

**Published:** 2021-12

**Authors:** Sharad Kharel, Archana Gautam, Manish Mahotra, Nasya Martin Theniko, Say Chye Joachim Loo

**Affiliations:** a School of Materials Science and Engineering, Nanyang Technological University, 50 Nanyang Avenue, Singapore 639798, Singapore; b Singapore Centre on Environmental Life Sciences Engineering (SCELSE), Nanyang Technological University, Singapore 637551, Singapore

**Keywords:** Okara, β-carotene, Micronutrient, Polysaccharide, Encapsulation, Bioavailability

## Abstract

Colossal amounts of food waste are generated and discarded daily at the expense of financial resources and at a detriment to the environment. One such food waste, okara – a soybean by-product, is valorized in this study by upcycling it into nutritional extracts for micronutrients encapsulation. Micronutrient malnutrition, particularly in the developing world, is a major public health challenge. Herein, okara extracts were obtained through a low-cost extraction process and was subsequently developed as an encapsulant material for micronutrients β-carotene, and ferrous sulphate encapsulation, using zein as an excipient. Spray-drying, as a scalable technique, was employed to produce various formulations which were assessed for release profiles, shelf-life, β-carotene antioxidant activity and cell cytotoxicity. Finally, an optimized dual-micronutrient formulation displayed a sequential release with ferrous sulphate releasing in simulated gastric fluid, and β-carotene releasing predominantly in simulated intestinal fluid. This sequential release profile favors the absorption of both the micronutrients and could potentially enhance their bioavailability.

## Highlights

• Okara was upcycled into encapsulation materials using microwave assisted technique.
• Okara and zein as excipient used as encapsulation substrate for micronutrients.
• The formulation produced desirable sequential release of micronutrients in SGF and SIF.

## Graphical abstract

**Figure:**
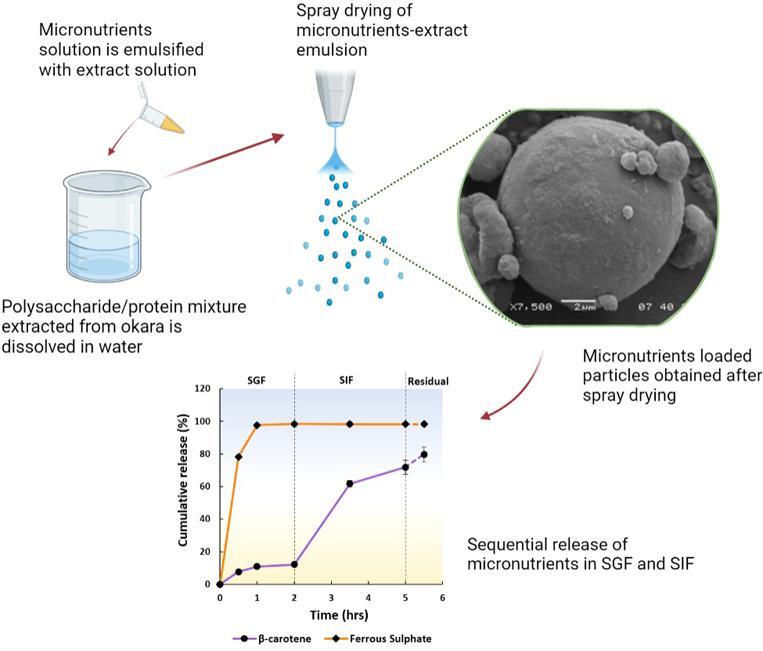

## 1 Introduction

More than two billion people in the world today are affected by micronutrient malnutrition (MNM) (2018). Vitamin A and iron deficiencies are the two of the most common forms of micronutrient malnutrition. These are more prevalent in children, women, and elderly of predominantly low-income countries in Asia and Africa (2018). Micronutrient deficiencies form an important global health issue, with malnutrition affecting key development outcomes including poor physical and mental development in children, vulnerability or exacerbation of disease, mental retardation, blindness, and general loss in productivity and potential (Ritchie & Roser, 2017). The World Health Organization (WHO) estimates that one third of the world population suffer from micronutrient deficiency globally (Bhutta et al., 2013). Most of the efforts to control the deficiency of these micronutrients generally use expensive supplementation strategies (Dairo & Ige, 2009). Although supplementation is necessary for groups at high risk and as a short-term emergency measure, a more effective, sustainable, and cost-effective nutritional intervention strategy that can be easily adapted into functional foods is required. Food fortification – the addition of micronutrients to common staple foods to maintain or improve the quality of one’s diet, can be a promising and viable approach to address MNM. However, micronutrients being labile in nature degrade rapidly and must be protected through micro-encapsulation for food fortification (Zimmermann & Windhab, 2010).

Vitamin A and iron are vital for the human body for proper immune function, growth, and development. The deficiency of vitamin A results in impaired immunity and haematopoiesis, xerophthalmia, and night blindness. Likewise, lack of iron causes anaemia. The human body, however, is incapable of producing these micronutrients de novo which warrants an external dietary intake. β-carotene (BC), one of the many provitamin A carotenoids, has the highest vitamin A activity and the most efficient conversion to vitamin A (Yeum & Russel, 2002). Furthermore, BC also has antioxidant capabilities which makes it an ideal candidate for vitamin A supplementation (Grune et al., 20102010). However, the bioavailability of BC from natural sources is poor due to the resistance of plant cell walls and carotene-protein complexes to digestion making it difficult to achieve the recommended intake of 2–4 mg/day (Rein, 2013). This has led to exciting opportunities to extract and isolate BC for delivery either as supplements or in fortified food matrices to improve bioavailability and achieve optimal health. Pure BC is a labile compound easily degraded by heat, light, and oxygen (Gul, 2015) and is also reported to rapidly degrade in the gastric phase during digestion (Cutting, 2011, Donhowe et al., 2014), which warrants for its encapsulation to protect it from degradation and premature release in the gastric condition. Likewise, iron may also be protected against deterioration by encapsulation. It can protect the iron from oxidation avoiding the unpleasant odours, tastes, and appearances. In addition, it shields the iron from interactions with inhibitors that reduce its bioavailability due to chelation.

Spray drying is a popular method for encapsulation because it is cheap, fast, scalable and has high

Reproducibility (de Vos et al., 2010), contributing to its widespread use and acceptance within the food industry for applications, e.g., encapsulation of enzymes, flavours, antioxidants, preservatives, and bioactives, to name just a few (Gibbs et al., 1999). Careful considerations must however be placed in selecting the right encapsulating matrix for spray drying. Numerous biopolymers have been exploited as encapsulating matrix for applications in food. Tapioca starch (LOKSUWAN, 2007), modified food starch (Liang et al., 2013), gum Arabic (Leach et al., 1998), cyclodextrin (Arikan & Rodway, 2000), maltodextrin (Desobry et al., 1997) have all been used to varying degrees of success. In contrast, iron is generally encapsulated in hydrogenated palm oil (Biebinger, 2009, Wegmüller et al., 2006) and soybean oils (Zlotkin et al., 2001) by spray drying. As the encapsulating material constitutes the bulk of the product, it contributes majorly to the cost of the product. As such, there is a continuous pursuit in exploring new encapsulating material that is cheap and can be used sustainably with minimum environmental impact. One such befitting candidate is “okara”.

Okara is a food processing by-product that consists of the insoluble remains of soybean in the production of soy-based food products (Ullah, 2017), e.g., soy milk and tofu. Despite it being nutritionally rich in proteins, potassium, calcium, isoflavone, polysaccharides, vitamin B, fat soluble nutritional factors and antioxidants, huge quantities of these are predominantly disposed daily. Disposal of okara is done at the expense of financial resources and poses a significant problem to the environment because of putrefaction and emission of greenhouse gases. Japan alone disposes 800,000 tons of okara annually costing ∼ USD 15 million (Li, 2013). Thus, turning these nutritionally rich ‘food waste’ into nutrient encapsulation substrate for food fortification is not only an economically sustainable approach to address MNM, but also a profitable proposition for okara waste management.

In this study, raw okara directly obtained from the food industry were processed to yield the nutritionally rich polysaccharide and protein extracts of okara (OE). These extracts were exploited to co-encapsulate both micronutrients, i.e., BC and iron (in the form of ferrous sulphate (FS)), through spray drying. Zein, a biopolymer derived from corn, also was used as an additive, to optimize the formulation. (Li, 2020) The encapsulation properties and particle characteristics of different formulations containing co-encapsulated nutrients, their potential cell cytotoxicity, release kinetics and shelf lives were evaluated and hereby reported. While other natural biopolymers have been previously investigated for the encapsulation of micronutrients, the co-encapsulation of the BC and FS within the extracts of okara to achieve desirable sequential release of both micronutrients along the gastro-intestinal tract and in extended shelf life is an exciting avenue that remains largely unexplored, making this work of interest to the food and nutritional research community.

## 2 Materials and methods

### 2.1 Materials

Fresh okara was obtained from Mr. Bean – a soy product company, Singapore. Zein protein, BC and FS were purchased from Sigma Aldrich, Singapore. High performance liquid chromatography (HPLC)-grade deionized (DI) water, chloroform, isopropyl alcohol, ethyl acetate and acetonitrile were used as solvents for extraction and analysis. The release media was prepared using pancreatin and pepsin enzymes, and Tween 20 purchased from Sigma Aldrich, Singapore.

### 2.2 Methods

#### 2.2.1 Extraction of polysaccharide and protein from okara

Okara polysaccharide and protein were extracted using a previously reported microwave-assisted water extraction technique (Tsubaki et al., 2009). Briefly, raw okara was defatted using hexane and dried overnight. The okara was suspended in pH-adjusted water and the soluble components of the okara were extracted by heating in a microwave oven. The resultant cooked mixture was centrifuged and filtered to remove the solid residue. This solution was then processed in two ways: 1. removing protein from the solution by precipitation, followed by centrifugation, and spray drying of the isolated extracts solutions to yield dry powder of polysaccharide and protein separately or, 2. spray drying the extract solutions directly to yield dry powder mixture of protein and polysaccharide extracts of okara. The extracts were then kept in airtight bags and stored at room temperature.

#### 2.2.2 Fourier transform infrared spectroscopy (FTIR)

FTIR analysis was done to identify polymer composition within the okara extracts. All the spray dried powder samples were thoroughly mixed with KBr powder and pressed into 13 mm pellets. The infrared spectra of pure KBr were set as the background of all the samples. Each of the sample’s spectrum was then recorded from wavenumber 4000 to 400 cm^−1^ with a phase resolution of 4 cm^−1^. Similarly, the FTIR spectra of the zein, BC and FS were also obtained as controls for FTIR analysis of the dual-polymer formulation loaded with both the micronutrients.

#### 2.2.3 Encapsulation of micronutrients

Two micronutrients, i.e., BC and FS, were encapsulated in the OE using a Mini Spray Dryer B-290 (Buchi). The formulations consisted of batches loaded with either one of the micronutrients, or both concurrently, fabricated using either okara-extracts alone, or in combination with another biopolymer like zein: a corn-derived protein which is resistant to dissolution in SGF. Additionally, the formulations were prepared by spraying from either single or double channels of a 3-fluid concentric nozzle (3N). All the formulations prepared are listed in Table 1**.**

**Table 1:** Table of spray-dried formulations using okara and zein loaded with micronutrients.

		Solution A Zein Solution		Solution B BC solution		Solution C OE solution		Spray Dry	
Formulation	Description	Conc. (mg/mL)	Vol. (mL)	Conc. (mg/ml)	Vol. (mL)	Conc. (mg/mL)	Vol. (mL)	Center channel	Peripheral channel
*F.1*	Okara (BC)	–	–	5	2	10	14	B-in-C emulsion	–
*F.2*	Zein (BC)	12	5	5	2	–	–	B-in-A emulsion	–
*F.3*	Okara (FS)	–	–	–	–	10	14	FS in C	B^a^
*F.4*	Zein (FS)	12	16.66	–	–	–	–	FS in A	A^a^
*F.5*	Okara + Zein (BC)	12	5	5	2	10	14	Blend of B-in-C emulsion with A	–
*F.6*	Okara-Zein (BC)	12	5	5	2	10	14	B-in-A emulsion	C
*F.7*	Zein-Okara (BC)	1.875	32	5	2	10	14	B-in-C emulsion	A
*F.8*	Zein (FS)-Okara (BC)	1.875	32	5	2	10	14	B-in C emulsion	A^a^

Denotations:

x-y represents x sprayed from peripheral and y is sprayed from center channels, of a 3-fluid concentric nozzle.

+ represents a blend of encapsulants x and y.

(z) represents the ‘z’ as the agent encapsulated in the encapsulant material.

^a^ solution A contains 10 mg FS.

* Encapsulation efficiency of FS.

#### 2.2.4 Spray solutions preparation

Briefly, zein was dissolved in alkaline water (pH 12), BC was dissolved in chloroform, and okara extract was dissolved in water to form solutions A, B and C respectively of various concentrations. Various emulsions were prepared which includes, emulsion of solution B-in-A (e.g., F.2, F.5 and F.6), or emulsion of solution B-in-C (e.g., F.1, F.7 and F.8). These emulsions were prepared over an ice bath using an ultrasound probe sonicator operated at 50% amplitude for 1 min with 5 s on and off pulse. At instances of FS encapsulation, FS is simply dissolved in solution A (e.g., F.4 and F.8) or solution C (e.g., F.3).

#### 2.2.5 Spray drying of the solutions

**Fig. 1:**
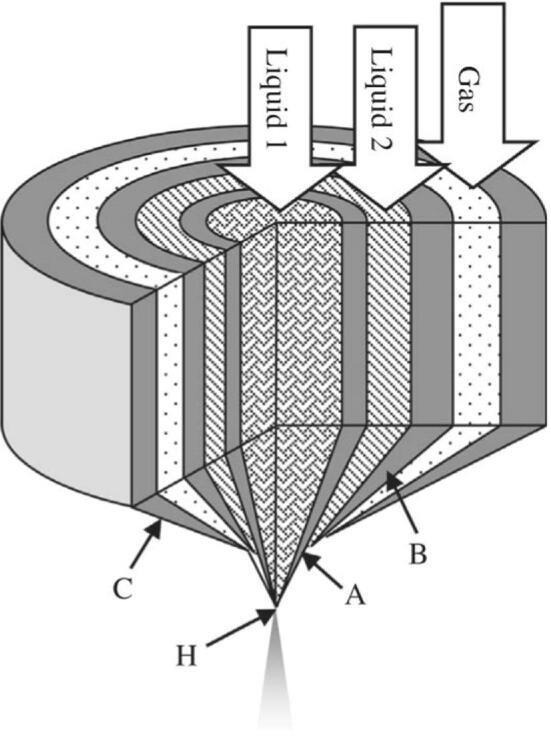
Schematic diagram of a three-fluid nozzle, (A) center nozzle, (B) peripheral nozzle, (C) air nozzle (3N), (D) liquid nozzle, (H) collision point.^1.^

The 3 N nozzle has a unique, three-layered concentric structure that consists of a center nozzle (inner liquid passage), a peripheral nozzle (outer liquid passage), and an air nozzle (outer- most gas passage) (Fig. 1). Two spray fluids are individually provided via the center and peripheral nozzles. The spray fluid provided via the peripheral nozzle is accelerated on the outside of the center nozzle by compressed air supplied from the air nozzle. The accelerated spray fluid and the other spray fluid provided via the center nozzle collide and are mixed at the tip of the center nozzle and are then atomized by compressed air. Atomized mist is dried with heated air to form composite particles, which are deposited at the collection bottle.

As such, these emulsions were immediately fed through the center channel of the spray dryer at 1.5 mL/min feed rate using an external peristaltic pump. Wherever applicable (see Table 1), additional solutions A or B were concurrently fed through the peripheral channel at 3 mL/min feed rate. For dual biopolymer formulations, the mass ratio of okara extract and zein were kept at 70:30 (okara: zein) for all such formulations. The rest of the spray drying parameters were set as: 150 °C inlet temperature, 70–90 °C outlet temperature, 40 q-flow. With these optimized parameters, free flowing dry powder were obtained in the collection bottle which were retrieved and stored at −20 °C until further tests.

#### 2.2.6 Morphological analysis

The particle morphologies and sizes of all the samples were analysed using a Field emission Scanning Electron Microscope operated at 5 KeV (FeSEM 7600F). Samples were uniformly spread onto metal stubs, finely coated with platinum using a sputter coater model SPI-Module and imaged by the FeSEM. ImageJ software was used to analyse the SEM images for particle size distribution.

#### 2.2.7 Determination of encapsulation efficiency of micronutrients

2 mg of each of the formulations was dissolved in 1 mL fed state simulated gastric fluid (FeSSGF, pH 5) with 0.1% Tween 20 (SGF) in an Eppendorf tube using ultrasound agitation for 5 min and occasional vortexing in between. 0.8 mL of this solution was then vortexed with 0.5 mL of chloroform and centrifuged at 5000 rpm for 3 min resulting in dissolution and partitioning of the BC in the lower chloroform fraction and FS in the upper aqueous fraction. The lower fraction was then analysed by HPLC and upper fraction by ICPMS to quantify the amounts of BC and FS, respectively. Encapsulation efficiency was calculated using the following equation:

$$\mathrm{Encapsulation}\;efficiency\left(\%\right)=\frac{\mathrm{measured}\;amount\;of\;micronutrient\;in\;sample}{\mathrm{theoretical}\;amount\;of\;micronutrient\;in\;sample}\times 100$$

#### 2.2.8 Release study of the micronutrients

2 mg of each formulation was suspended in 1 mL FeSSGF in a shaking incubator at 37 °C. At each predetermined time points (0.5 h, 1 h, 2 h, 3.5 h and 5 h), the samples were centrifuged at 5000 rpm for 3 min. Subsequently, 0.8 mL of the supernatant was collected for analysis and replenished with 0.8 mL of fresh media. The media was changed to simulated intestinal fluid (SIF, pH 6.8) with 0.1 % Tween 20 (SIF) at 2 h time point and continued henceforth in the same media. After media collection at each time point, the samples were briefly vortexed to redistribute the particles in the media before putting the samples back in the incubator. The collected samples of all the time points were further processed to extract the BC that was released. For this, 0.8 mL of each of the samples were briefly vortexed with 0.5 mL chloroform and centrifuged at 5000 rpm for 3 min. Subsequently, 200 μL of the lower chloroform fraction was collected and analysed using high performance liquid chromatography (HPLC). In contrast, 0.5 mL of the upper aqueous fraction was analysed using an inductively coupled plasma optical emission spectrometer (ICP-OES) to quantify the amount of the FS released in the medium. At the end of release study, the residual unreleased BC and FS were also retrieved and quantified.

#### 2.2.9 Quantification of BC using HPLC and FS using ICP-OES

The samples were measured in Agilent 1100 series HPLC module using a BC-Zorbax SB-C18 Analytical (4.6 × 250 mm, 5 μm) chromatographic column maintained at 25 °C. The mobile phase comprised of acetonitrile, isopropyl alcohol, and ethyl acetate (40:40:20) at a flow rate of 2 mL/min in isocratic mode. The sample injection volume was 10 μL and the effluent was monitored at 448 nm. Each time, the mobile phase was freshly prepared before use. Freshly prepared solutions of BC in chloroform of known concentrations (0.001–0.1 mg/mL, n = 3) were measured and plotted against the total peak area of the BC to prepare the calibration curve. The cumulative amount of BC was calculated using the calibration curve and plotted against the time points to obtain the release profile.

The Fe content in the release media were analysed using a Perkin Elmer Optima 8300 inductively coupled plasma optical emission spectrometer (ICP-OES). This was then correlated with the actual amount of FS released in the media from the particles. All calibration curves produced correlation coefficients >0.99.

#### 2.2.10 Antioxidant activity of β-carotene as a measure of its bioactivity

The antioxidant capacity of the encapsulated BC (eBC) of all the formulations were evaluated against the unencapsulated pristine BC (uBC) using ABTS radical scavenging assay. (Barton, 2010) Briefly, 58.33 mg of sodium persulfate (SF) was dissolved in 0.1 L of 0.01 M PBS solution to form 2.45 mM solution of sodium persulfate. After, 0.3841 g of ABTS was dissolved in the SF solution and then set aside for conditioning overnight in darkness at room temperature.

Next day, the formulations were processed for analysis under two different conditions: 1. without interaction with FeSSGF, and 2. after 3 h exposure to SGF. Weighted amounts of each formulation were suspended in FeSSGF for 3 h and the eBC was subsequently extracted in chloroform. Extraction process involved adding chloroform to the suspension, repeated cycles of vigorously vortexing and ultrasound agitation at 70 °C, and finally centrifuging at 10 k rpm after which the lower chloroform fraction was collected. The chloroform was then removed by vacuum drying and precipitated BC was reconstituted in DCM. On the other hand, BC was also similarly extracted and reconstituted in DCM from all the formulations instantly, without subjecting them to the 3 h of exposure to FeSSGF. Resultant BC solutions were measured using HPLC and necessary dilution was done with DCM to normalize the concentration of each sample solution to the working concentration of 0.07 mg/mL. After this, 1 mL of the overnight conditioned ABTS stock solution was diluted in 40 mL DI water to obtain the absorbance of 0.703 ± 0.005 units at 734 nm using the cell plate reader. 60 μL of each of the extracted BC samples were then mixed with 940 ul of this diluted ABTS solution. The mixture was vigorously vortexed, centrifuged and 150 μL of upper DCM fraction was added in each well of 96 well plate and immediately measured at 734 nm. Fresh eBC solution in DCM and blank DCM served as positive and negative controls. Results were expressed as % of the scavenging effect of pristine uBC.

#### 2.2.11 Shelf-life evaluation of encapsulated β-carotene

Shelf-lives of all the formulations were evaluated. For this, equal weighted amounts of all the formulations along with pristine uBC and spray-dried uBC were put into vials and stored at N_2_ glove box protected from light, humidity, and air. In contrast, another set of samples were kept outside exposed to open air, ambient light, and moisture. At predetermined intervals, 2 mg of each sample is retrieved and the eBC is extracted from each sample using as previously described. As control, uBC was subjected to a similar extraction process. Finally, the extracted BC from all the formulations is measured on HPLC using the method discussed earlier. A graph is plotted, with y-axis showing the % of relative change in the amount of intact BC over time.

## 3 Result and discussion

### 3.1 Upcycling of okara as an encapsulant material

This paper aims to converge two global issues into a food nutrition solution. First, is the colossal amount of food waste generated and discarded daily, at the detriment to the environment and at the expense of financial resources. Second, a vast proportion of the global population suffers from micronutrient malnutrition (i.e., vitamins, iron and iodine) with devastating consequences to health and the economy. (World Health Organization, 2001, Zimmermann, 2004) These two challenges however present a tremendous opportunity whereby the waste from the food industry could be valorized into a nutritionally-enriched substrate that can be upcycled for micronutrient encapsulation. Encapsulation of these micronutrients not only protects labile compounds from degradation but can potentially improve bioavailability through controlled, targeted gastrointestinal release, while prolonging shelf-life. Additionally, such formulations permit their incorporation into food by improving heat stability and masking of any unsavoury tastes.

While it would be desirable to use the whole composition of raw okara, its insoluble components are unsuitable for nutrient encapsulation. Thus, soluble okara compounds were first extracted through a modified microwave-assisted extraction technique for better efficiency, scalability, and cost effectiveness. This process enables the extraction of soluble okara components at a high temperature. The insoluble residue was subsequently filtered out. At this stage, previously reported works employed extraction of the polysaccharides and proteins from the solution by precipitating in a co-solvent like ethanol, 3–5 times the volume of the solution (Furuta et al., 1998). Instead, by employing industrially scalable spray drying, dried powder of okara extracts can be rapidly obtained sans secondary solvents. This method is faster, more economical with a higher throughput than earlier reported solvent extraction techniques (Furuta et al., 1998). Given the processability of the extracts at this stage, no further purification is needed before encapsulation of the micronutrients.

**Fig. 2:**
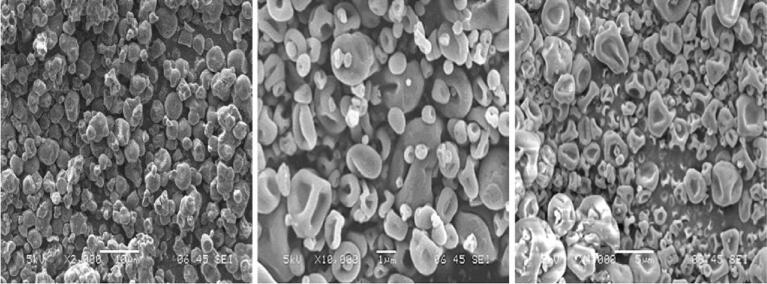
FeSEM image of spray dried powder of **a.** mixture of okara polysaccharide and protein extracts, **b.** okara polysaccharide extract and **c.** okara protein extract.

The yield of the extracts varied significantly with different processing and spray-drying parameters. A total maximum extract yield of 61.6 ± 24% was obtained that yielded a mix of polysaccharide and protein. Henceforth, the mix extract of polysaccharide and protein concurrently extracted under aqueous conditions were used for the rest of the studies. Three different batches were produced which are polysaccharide extracts, protein extracts and mixture of the formers. FeSEM images of the samples are shown in Fig. 2. The polysaccharide and protein extract powders showed similar deflated particle morphologies. In contrast, the batch composing of a blend of protein and polysaccharide extracts showed comparatively denser looking spherical particles.

**Fig. 3:**
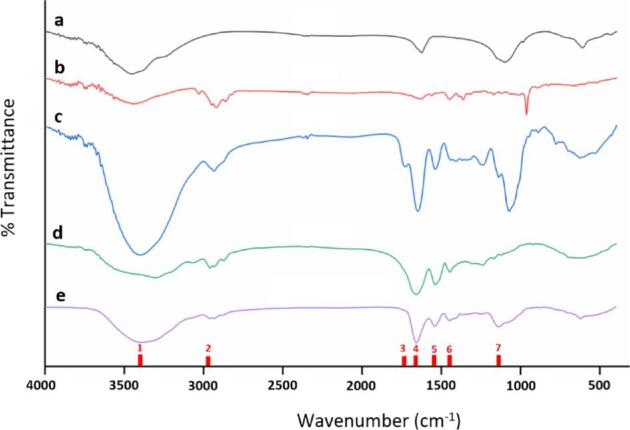
Image showing FTIR spectra of the FS (a), BC (b), OE (c), zein (d), and formulation F.8 (e).

The mix batch was analysed as a representative batch using FTIR for confirmation of the polysaccharide and protein composition of the powder (Fig. 3). The broad stretched strong peak observed at 3400 cm^−1^ (denoted with ‘1’), a weak band at around 2930 cm^−1^ (denoted with ‘2’) and a peak around 1740 cm^¬1^ (denoted with ‘3’) are the characteristic absorption of O—H bond, C—H bond, and carboxylic ester bond, respectively in polysaccharide molecules. On the other hand, the amide I bond and amide II bond at 1650 cm^−1^ and 1550 cm^−1^ (denoted with ‘4’ and ‘5’ respectively) indicative of protein.

### 3.2 Micronutrients encapsulation into okara extracts by spray drying

BC, a precursor to vitamin A and FS (iron) were chosen as the micronutrient candidates as they have been reported to be the key micronutrients lacking in diets of population suffering from MNM. While BC is the most potent precursor of vitamin A, FS is one of the lowest costs of iron supplements and also reported to have higher bioavailability as compared to other forms of iron. BC is a fat soluble hydrophobic compound, whereas FS is a highly water-soluble compound.

Three different categories of formulations were fabricated: 1. Single biopolymer-single micronutrient (F.1–4), 2. dual biopolymer-single micronutrient (F.5–7) and 3. dual biopolymer-dual micronutrients (F.8). For the latter two categories of formulations, another biopolymer zein was also used in combination with the extracts of okara. While okara extracts remain the predominant substrate (70%), the amount of zein used was limited to only 30% of the total material composition. This ratio was carefully selected to keep the amount of zein in the formulation at its lowest while still ensuring effective encapsulation. The solution feed rate for the inner and outer channels of the spray dryer was kept at 1:2 to allow for the proper coating of the inner channel solution droplets by the outer channel solution. With this in mind, the volume and concentration (amount) of the inner and outer channel solution were optimized.

**Fig. 4:**
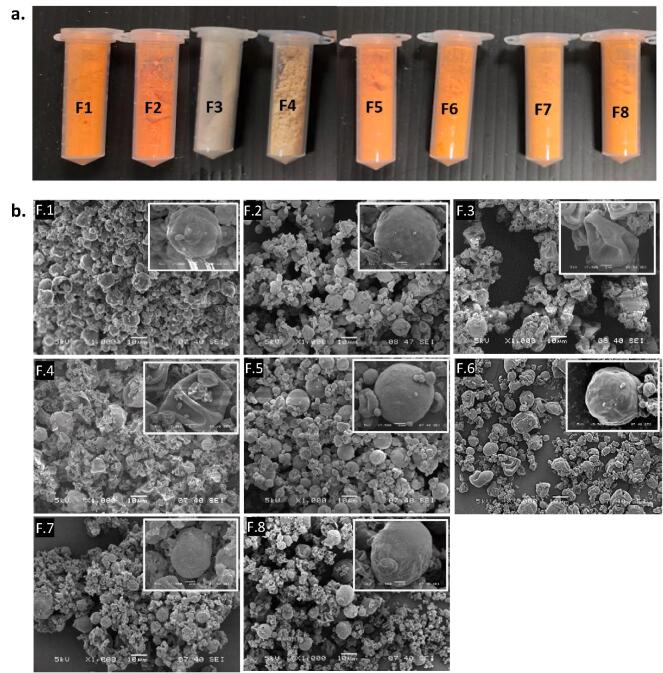
Figure showing **a.** a series of micronutrient loaded formulations prepared for the study, **b.** FeSEM image of the micronutrient encapsulated particles.

All formulations loaded with BC visually appeared bright orange (Fig. 4a), irrespective of whether OE and/or zein were used as encapsulants. FeSEM image of the formulations showed variable size distribution and morphology of particles, whereby F.3 and F.4 resembled a deflated balloon morphology, the rest of the formulations were spherical particles and seemingly denser (Fig. 4b**)**. As all the formulations were spray dried with the same parameters, the reason for this variation in morphology could be attributed to the difference in the processing steps of the solution prior to spray drying. While for the rest of the formulations, the BC solution was emulsified with the okara or/and zein solution using ultrasound probe, this step was not employed for F.3 and F.4 (i.e., no BC) as FS readily dissolved in the encapsulant solutions.

The encapsulation efficiency was observed to vary significantly among the formulations. From Table 2, it is evident that EE is highest when a single micronutrient was encapsulated in a single biopolymer formulation. In such formulations, the EE ranged from 35 to 57% (F.1-F.2) for BC and 54 to 65% (F.3-F.4) for FS. In contrast, encapsulation of BC in dual polymer systems generally reduced the EE, resulting in 26% and 33% EE for F.5 and F.7, respectively. Interestingly, having BC incorporated in the zein solution rather than the okara solution in dual polymer formulations retained higher encapsulation of BC (58%) for F.6. This perfectly matches the EE of F.2 where BC was also incorporated in zein. It can thus be deduced that zein aids in better encapsulation of BC which may be due to its amphiphilic nature and film-forming properties (Li, 2020). Lastly, the EE of BC is significantly reduced to 21% (F.8) when both FS and BC are concurrently encapsulated in a dual polymer formulation. Considering F7 and F8 together, it can be deduced that simultaneous encapsulation of FS negatively affected the EE of BC. In a nutshell, the incorporation of an additional polymer or micronutrient decreases the EE of BC in general.

**Table 2:** Encapsulation efficiency of BC and FS in different formulations.

Formulations	Description	BC Encapsulation Efficiency (%)	FS Encapsulation Efficiency (%)	Yield (%)	Remarks
F.1	Okara (BC)	34.6 ± 9.3	–	83	
F.2	Zein (BC)	57.2 ± 1.4	–	80	Single biopolymer formulations loaded with single micronutrient
F.3	Okara (FS)	–	53.8 ± 9.2	66	
F.4	Zein (FS)	–	64.6 ± 3.3	76	

F.5	Okara + Zein (BC)	26.3 ± 3.3	–	82	Dual biopolymer formulations loaded with single micronutrient
F.6	Okara-Zein (BC)	58.3 ± 6.8	–	81	
F.7	Zein-Okara (BC)	32.7.6 ± 0.3	–	72	

F.8	Zein (FS)-Okara (BC)	20.9 ± 0.3	67.6 ± 7.5	82	Dual biopolymer formulations loaded with two micronutrients

The presence of zein in the formulation was assessed by the FTIR analysis. The formulation F.8 was analysed as a representative formulation. The arising of a band at 1102 cm^−1^ (denoted with ‘6’), which also is a noticeable peak in the spectrum of zein (control) in the FTIR analysis, confirms the successful incorporation of zein in the formulation. This is further evident from the significant reduction in the intensity of the peak at 1072 cm-1 (denoted with ‘7’) observed in the F.8 spectrum in comparison to the OE (control) spectrum.

Overall, the fabrication yield was greater than 65% for all the formulations, peaking at 83% for F.1. Considering this high yield of the production, even for this low working amounts of polymer whereby a minor loss of the materials can translate into huge % yield loss, we envisage that this technique can be translated into a commercial scale process for micronutrients encapsulation.

### 3.3 Evaluation of release of micronutrients in the simulated digestive fluids

**Fig. 5:**
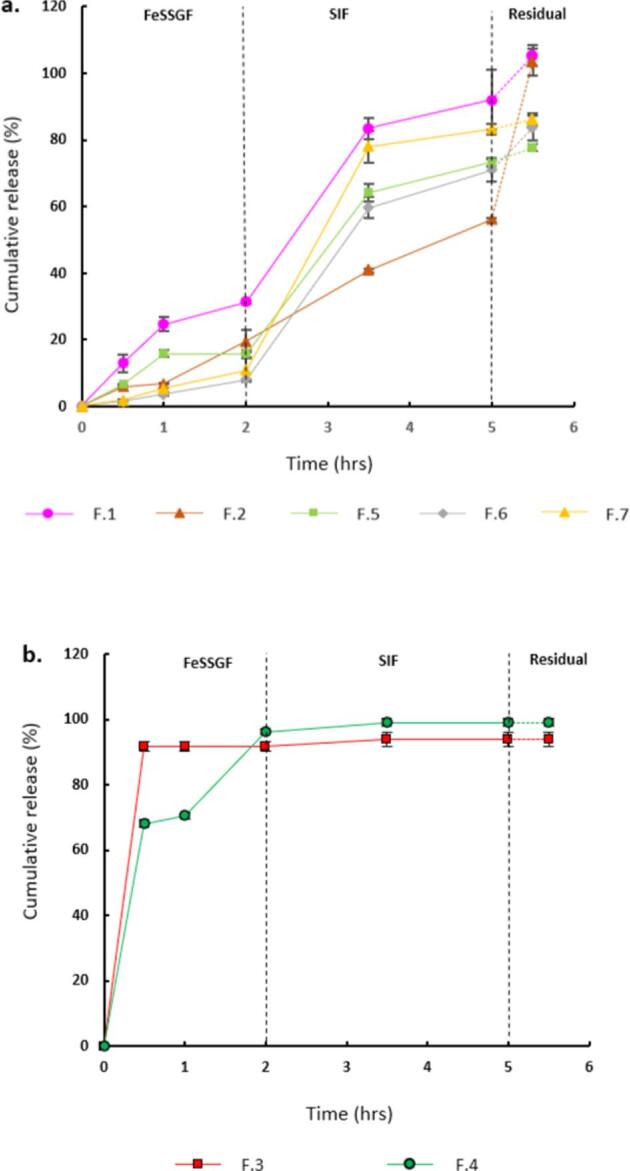
Graphs showing release profiles of **a.** BC and **b.** FS for all the formulations in FeSSGF (pH 5) for 2 h followed by additional 3 h in SIF (pH 6.8). The unreleased amount of BC is also quantified and plotted as residual.

As the formulation is envisioned to be incorporated into food, it is crucial to evaluate the release kinetics of these encapsulated micronutrients. For this, the formulations were tested sequentially in two different simulated digestive fluids, i.e., FeSSGF (2 h) and SIF (3 h), to simulate digestion. FeSSGF of pH 5 was specifically chosen for the reason that the formulations are intended to be consumed along with dietary food. Results showed that the release profile was greatly affected by the choice of encapsulant (Fig. 5). F.1 which is composed entirely of OE showed a comparatively higher release of 25 % and 31 % in 1 h and 2 h duration, respectively. This was followed by a steep increase in the release when the media was changed to SIF, reaching a cumulative release of 92 % in 5 h. In contrast, a slower release of 5 % and 20 % was observed over similar durations for F.2 which is composed entirely of zein. The highly crystalline and hydrophobic nature of zein (Li, 2020) explains this slower release observed in comparison to the OE particles. Further, the cumulative release amount was comparatively lower at 56 % in 5 h in SIF. The gradient of the release curve revealed that the pH of the release media had no effect on the release from F.2, unlike other formulations.

All the other batches that were composed of both the polymers showed a suppressed release in FeSSGF similar to F.2 and a drastically higher cumulative release in the SIF. In essence, F.7 was found to have the most desirable release profile. On the contrary, complete release of FS was observed in FeSSGF for the FS loaded formulations. F.3 formulation released all of its payload in the very first 0.5 h, while F.2 released 68% of its payload at the same time ultimately reaching 100% in 2 h exposure in FeSSGF. Taken together, two different release profiles unique to BC and FS were observed from the formulations. While the release of BC was predominant in the SIF, FS is completely released in FeSSGF. Given the sensitivity of BC to SGF, this result is favourable as the final aim is to release BC in SIF to avoid its reported degradation in gastric environment.

### 3.4 Release study of co-encapsulated ferrous sulphate and β-carotene

**Fig. 6:**
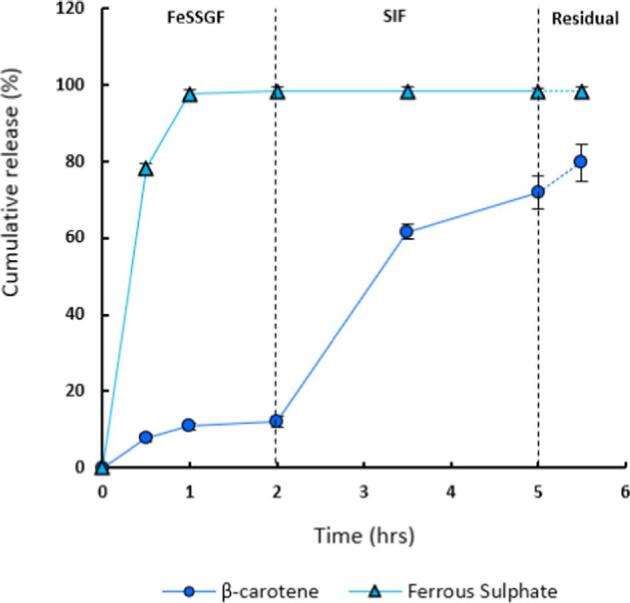
Graph showing release profiles of single formulation with co-encapsulated BC and FS (F.8), in FeSSGF for 2 h, followed by an additional 3 h in SIF. The unreleased amount of BC and FS is also quantified and plotted as residual.

FS and BC were subsequently co-encapsulated, and its release was conducted for this formulation (F.8). The release profile of BC observed a similar trend as reported above. FS released completely in FeSSGF in the first 2 h, with only 12 % of the BC released during this period. Subsequently, a more rapid release of BC (71 % in 5 h) in SIF was observed (Fig. 6). The preferential release of FS and BC in FeSSGF and SIF respectively is advantageous to improve bioavailability and due to the beneficial interactions of these micronutrients in metabolism (Zimmermann & Windhab, 2010). For instance, vitamin A deficiency may exacerbate anaemia through impairment of iron metabolism (Semba and Bloem, 2002, Maqsood, 2004). Co-encapsulation into a single carrier may also be simpler and more economical as a food additive.

### 3.5 Assessment of the antioxidant activity of β-carotene as a measure of its bioactivity

**Fig. 7:**
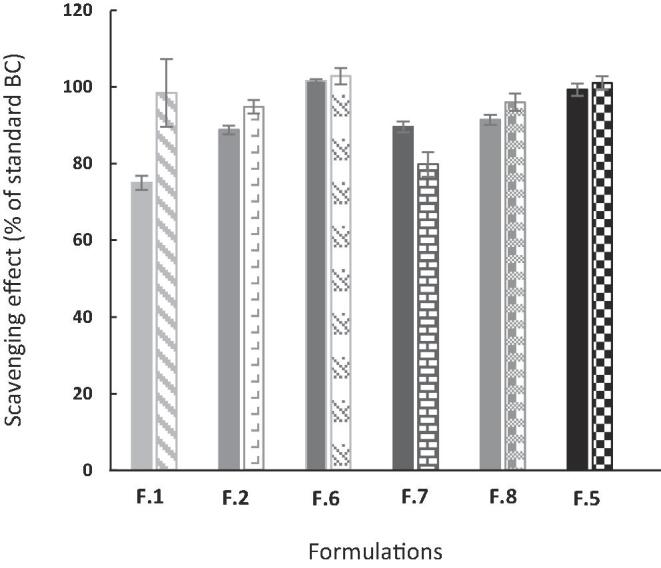
Result showing the radical scavenging effect of BC encapsulated in various formulation: without exposure in FeSSGF (checkered), and after 3 h exposure in FeSSGF (solid).

Several studies have shown that BC has lipid-soluble antioxidant activity and is effective in protecting against free radicals (Sánchez-Moreno et al., 2003, Boon et al., 2010). Accumulation of free radicals in the body is known to cause cellular and tissue damage due to oxidative stress leading to diseases such as cancer, heart disease and cognitive disorders like Alzheimer’s disease (Uttara et al., 2009). BC helps in combating these conditions due to its antioxidant properties (Uttara et al., 2009). Hence, it is imperative that its innate antioxidant activity remains unaltered after subjecting to the fabrication process. Antioxidant activity of the encapsulated BC was measured for all the formulations prepared and this was plotted as the % of antioxidant activity of uBC. The result showed that after encapsulation through spray drying, the antioxidant activity for most formulations, remains unremarkable (Fig. 7). However, a reduction of 20% of the antioxidant activity was observed in the F.4 for reasons currently unknown.

Further tests were conducted to ascertain whether the formulations can preserve the antioxidant activity of the BC upon 3 h of exposure to FeSSGF. The results show that the eBC retained its antioxidant activity even after exposure in the FeSSGF which indicates that the formulation can preserve the antioxidant activity of BC in a gastric environment. Specifically, the formulation containing co-encapsulated micronutrients not only showed desirable release kinetics of each micronutrient but also performed remarkably well in preserving the functionality of BC. These formulations were also analysed for any possible cell cytotoxicity and were confirmed to be safe for use in food (**supplementary information**).

### 3.6 Evaluation of shelf life of the β-carotene encapsulated formulations

Studies have confirmed that micronutrient fortification of foods can greatly aid in tackling the problem of micronutrient deficiencies. (Hurrell, 2002) However, micronutrient storage stability issues have greatly dwarfed the prospects as this issue remains largely unaddressed. Thus, the shelf-life of the formulations was investigated to highlight the potential of encapsulation in improving shelf-life. The uBC was included as a control to evaluate the outcome of encapsulation. Samples were tested at two different conditions: ambient environment vs inert environment. Ambient environment represents the worst scenario where the formulations are exposed to all the environmental factors like air, humidity, light, and moisture. On the contrary, an inert environment represents the best-case scenario where the samples are kept in an inert (N_2_) environment isolated from those environmental factors.

**Fig. 8:**
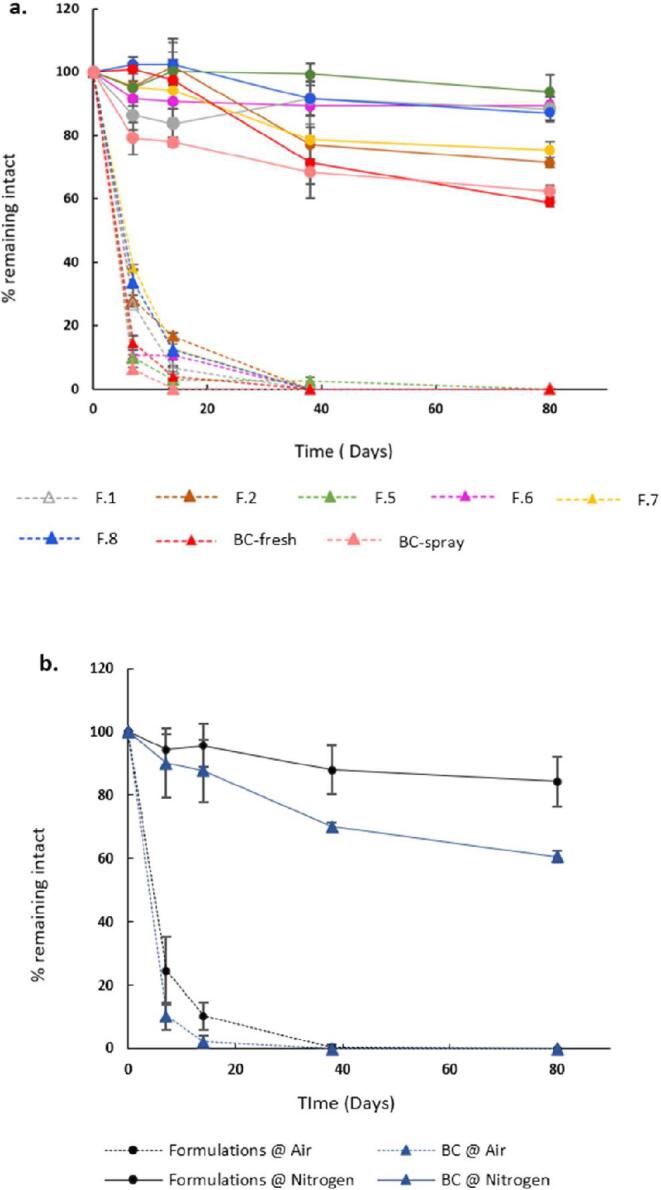
Results showing **a.** the shelf life of each formulation loaded with BC in inert (solid line) vs ambient (dotted line) conditions, **b.** the comparison of average of the shelf life of all the eBC formulations vs the uBC.

The results showed a broad distinction between the inert and ambient environment on the shelf-life of the formulation. While the formulations were stable (up to 80 days) in an inert environment over the long storage duration, formulations stored in ambient conditions degraded by 50–90% at day 7, reaching complete degradation by day 40 (Fig. 8a). F.5, in particular, was able to retain 100% of BC in the 80-day study period. One key observation common to both groups was that shelf-life of eBC was longer than uBC. This becomes evident from the second graph which shows the average value of all the formulations in comparison to uBC (Fig. 8b). The indication that eBC formulations performed better than uBC becomes increasingly evident by day 80 in the inert environment. While the % of intact BC ranged 82 ± 9% for the eBC, the level decreased to 60 ± 1.86% for uBC kept under the same environment. This implies that a minor level of BC degradation for the formulations is to be expected even in the ideal storage conditions over long storage duration. However, the BC-encapsulated formulations continue to perform comparatively better than the unencapsulated counterpart, further corroborating the protective effect imparted by the encapsulation technology used.

## 4 Conclusion

A scalable and cost-effective technique was devised to recycle the industrial food waste okara into high value nutritionally rich polysaccharide and protein extracts. These extracts were then upcycled into natural encapsulant materials for the micronutrients BC and FS, with zein as a formulation property enhancer. The formulations were screened for various attributes such as release profile and kinetics, shelf-life, antioxidant activity and cytotoxicity. With the preliminary knowledge of the formulations of individually encapsulated micronutrients, one single formulation with both micronutrients co-encapsulated was optimized and further characterized. This formulation displayed a desirable sequential release kinetics of the micronutrients, with FS releasing in FeSSGF and BC releasing into SIF, the respective favourable sites of absorption of both the micronutrients. The formulations were also shown to protect the bio-functionality of the micronutrient and comparatively improve the shelf-life as compared against pristine BC. Finally, the formulations were shown to be safe without any observed cytotoxicity.

## Ethical statement

The authors indicate that this research did not include any human subjects and animal experiments.

## CRediT authorship contribution statement

**Sharad Kharel:** Conceptualization, Methodology, Investigation, Writing – original draft, Writing – review & editing, Data curation. **Archana Gautam:** Writing – original draft, Methodology, Investigation. **Manish Mahotra:** Investigation, Validation. **Nasya Martin Theniko:** Investigation, Validation. **Say Chye Joachim Loo:** Conceptualization, Supervision, Writing – review & editing, Project administration, Funding acquisition.

## Declaration of Competing Interest

The authors declare that they have no known competing financial interests or personal relationships that could have appeared to influence the work reported in this paper.
