# Supplementary material for: Valorizing okara waste into nutritionally rich polysaccharide/protein-extracts for co-encapsulation of β-carotene and ferrous sulphate as a potential approach to tackle micronutrient malnutrition
Source: J Funct Foods. 2021 Dec;87:104749. doi: 10.1016/j.jff.2021.104749 (PMC8689405; doi:10.1016/j.jff.2021.104749)
Supplement: Supplementary Data 1 [file mmc1.docx]

## Assessment of cell cytotoxicity

The safety of the formulation was evaluated to ascertain its worthiness as a food ingredient. All the formulations were evaluated for cell cytotoxicity using the immortalized cell line of human colorectal adenocarcinoma (Caco-2). Briefly, Caco-2 cells were cultured in Dulbecco's Modified Eagle Medium (Gibco) supplemented with 10% FBS, 5% antibiotic and 4mM L-Glutamine at 37 °C and 5% CO2, until 70 to 80% confluence was reached. They were then trypsinized with 0.05% trypsin and collected by centrifuging at 250 g for 5 min. The cell pellet was resuspended in complete media and seeded into 48 well plates at the density of 4×105 cells.cm^-2^ and incubated for 24 h to ensure complete attachment of the cells. Stock solutions (1 mg/mL) of each formulation and fresh uBC (positive control) were prepared and diluted in complete medium into testing concentrations of 10 μg/mL and 100 μg/mL. Finally, the cell media was then replaced with the testing solutions and incubated for 24 h. Blank medium was used as a negative control.

Next, the cell media was replaced with 10% solution of alamarBlue (Invitrogen) reagent in serum free medium and incubated for 30 mins for the reaction to occur. Absorbance of the reagent was measured at 570 nm and 600 nm wavelengths with a microplate reader (Infinite M200, TECAN Inc., Mannedorf, Switzerland) which was then correlated with cellular metabolic activity according to the manufacturer's protocol. Similarly, for PicoGreen assay, after 24 h treatment, the cell media was removed, and the cells were washed thrice with PBS. The cells were lysed with a chilled cell lysis buffer (1× in DI water) (Cell Signalling Technology). The cell lysates were mixed with PicoGreen reagent according to the manufacturer's protocol and fluorescent reading was measured with the microplate reader.

The alamarBlue assay results showed that there was no indication of cytotoxicity for the formulations (**Fig.9**). The eBC formulations and uBC showed no remarkable reduction in the cellular metabolic activity at either of the concentrations. The data was also corroborated with the PicoGreen assay. The cell proliferation was mostly higher or equal to the negative control for most of the formulations which could be due to the carrier materials acting as the source of nutrients for the cells. These observations suggest that these formulations are potentially non-toxic and biocompatible and can be a promising encapsulant material for micronutrients. Interestingly, at the higher concentration of 100 μg/mL, unlike the micronutrient encapsulated formulations, the uBC showed a significant drop in the cell viability, which could be an impetus for encapsulating BC.


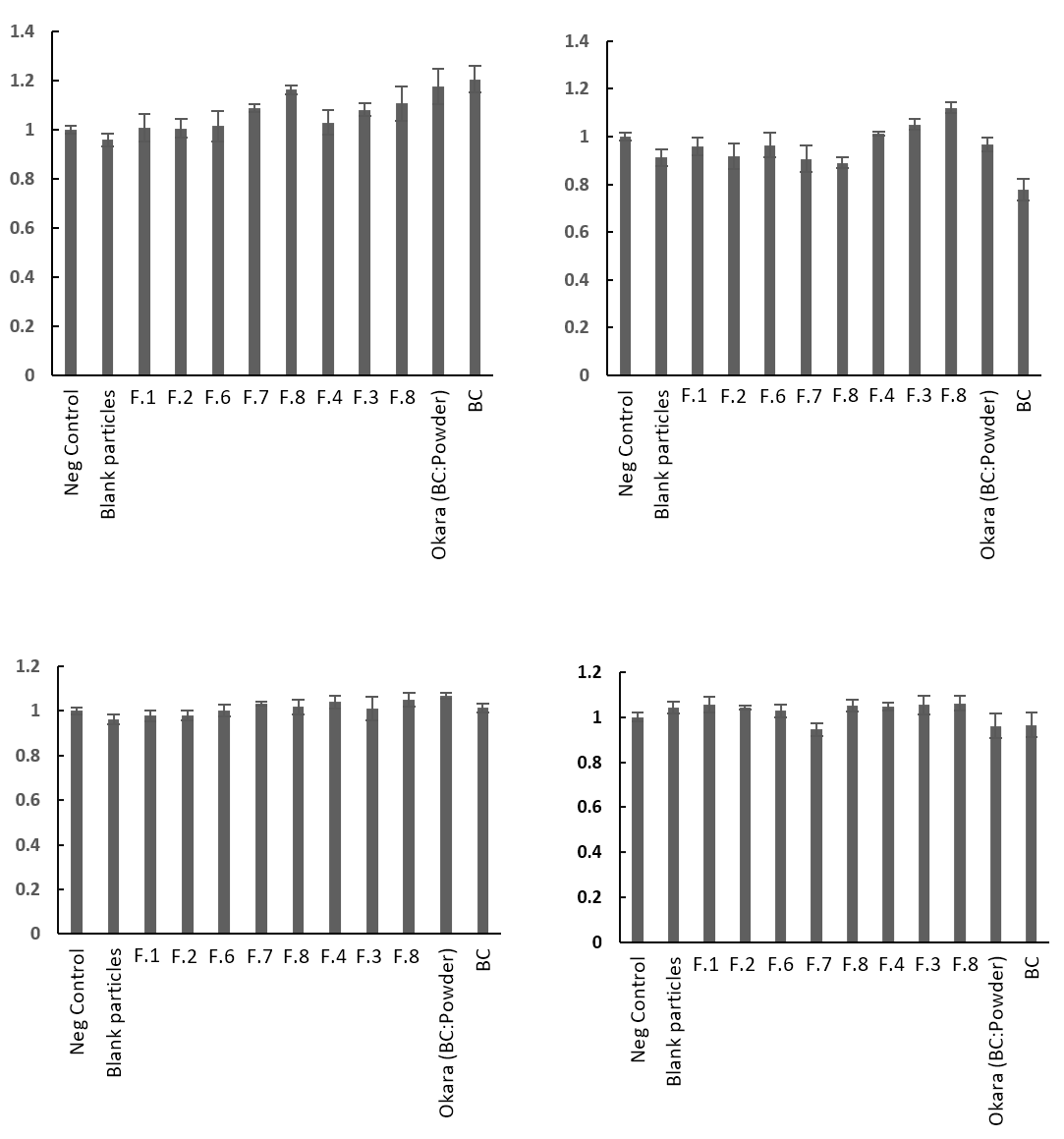


**b.**

**a.**

**Fig.9.** Results showing the cytotoxicity effect of the formulation using **a.** PicoGreen assay and **b.** alamarBlue assay (left: 10 μg/mL, right: 100 μg/mL concentrations)
